# The Potential Mechanism of Cuproptosis in Hemocytes of the Pacific Oyster *Crassostrea gigas* upon Elesclomol Treatment

**DOI:** 10.3390/cells14030199

**Published:** 2025-01-29

**Authors:** Yuxin Zhang, Jiejie Sun, Shurong Li, Lingling Wang, Linsheng Song

**Affiliations:** 1 Liaoning Key Laboratory of Marine Animal Immunology & Disease Control, Dalian Ocean University, Dalian 116023, China; zhangyuxin0006@163.com (Y.Z.); smokingcity@163.com (S.L.); wanglingling@dlou.edu.cn (L.W.); lshsong@dlou.edu.cn (L.S.); 2 Liaoning Key Laboratory of Marine Animal Immunology, Dalian Ocean University, Dalian 116023, China; 3 Dalian Key Laboratory of Aquatic Animal Disease Control, Dalian Ocean University, Dalian 116023, China; 4 Laboratory of Marine Fisheries Science and Food Production Processes, Qingdao National Laboratory for Marine Science and Technology, Qingdao 266235, China; 5 Southern Marine Science and Engineering Guangdong Laboratory (Zhuhai), Zhuhai 519000, China

**Keywords:** cuproptosis, elesclomol, hemocytes, protein lipoylation, *Crassostrea gigas*

## Abstract

Cuproptosis is a novel cell death dependent on mitochondrial respiration and regulated by copper. While the study of it is mainly focused on tumor therapy, in the present study, two key cuproptosis-related genes, ferredoxin (*FDX1*) and dihydrolipoamide S-acetyltransferase (*DLAT*) homologs (designated as *CgFDX1* and *CgDLAT*), were identified from *Crassostrea gigas*. *Cg*FDX1 has a Fer2 domain with a 2Fe-2S cluster forming a unique ferredoxin. *Cg*DLAT is composed of a biotin_lipoyl domain, an E3-binding domain, and a 2-oxoacid_dh domain. *CgFDX1* and *CgDLAT* mRNA were expressed in all the examined tissues. After elesclomol treatment, both mRNA and protein expressions of them were reduced in the hemocytes. The mortality rate of the hemocytes increased significantly, and the hemocytes were accompanied with noticeable adhesive abnormalities and heightened secretion after elesclomol treatment. Additionally, the accumulation or depletion of actin was observed in the hemocytes. The integrity of the double membrane structure of the mitochondria was compromised, and the organization of mitochondrial cristae was disrupted. The contents of copper, malondialdehyde (MDA), pyruvic acid and mitoSOX as well as the ratio of cells with low mitochondrial potential increased significantly in the hemocytes upon elesclomol treatment and the content of citric acid decreased significantly. These findings suggest the potential presence of cuproptosis in oysters and its activation mechanism is relatively conserved in evolution.

## 1. Introduction

Cuproptosis, a novel and distinct form of cell death, is characterized by its unique induction through copper dysregulation [1]. Cuproptosis induced by excessive copper accumulation disrupts the aggregation of dihydrolipoamide S-acetyltransferase (DLAT) and the stability of iron-sulfur (Fe-S) cluster proteins [2]. This contributes to the disruption of the tricarboxylic acid cycle (TCA cycle) and rupture of the cell membrane or deformation of the cell vacuole, which result in proteotoxic stress and eventual cell death [3,4].

Cuproptosis occurs through direct binding of copper to lipoylated components in the TCA cycle, leading to the aggregation of the copper-bound lipoylated mitochondrial proteins and a reduction in Fe-S cluster proteins [1,5]. Till now, cuproptosis has been reported in humans, mice, chickens, and some plants [6,7]. The activation of cuproptosis is linked to a downregulation of genes involved in redox metabolism regulation, such as ferredoxin 1 (*FDX1*), *DLAT*, etc. FDX1 located in the mitochondria, plays a crucial role in providing electrons for the free-radical chain reaction catalyzed by thioctanoyl synthase [8]. DLAT is an essential regulatory protein in mitochondria, which links the glycolytic pathway to the oxidative pathway of the TCA cycle [9].

Copper as a critical element in marine biogeochemistry, exhibits both bioavailability and toxicity [10]. It serves as an essential metal cofactor in biological enzymes, participating in various physiological processes [11]. Bivalves, as typical filter-feeding organisms, respire and ingest via their gills. They are capable of enriching copper within their bodies from the seawater [12]. Copper exposure could enhance the antimicrobial capacity of molluscan hemocytes by modulating phagocytic lysosomal processes, interfering with oxidative phosphorylation and inducing mitochondrial reactive oxygen species (ROS) production [13]. Oyster hemocytes are the primary carriers of transported and detoxified metals. They are chelated by copper chaperones via metal transporters translocating in the cytoplasm and also chelate copper into membrane-bound particles via endocytosis [14]. These indicate the serious impact of copper exposure on bivalves’ health by affecting respiratory metabolism and oxidative stress.

The intracellular copper levels are regulated by the action of copper uptake protein 1 (CTR1) and copper transporter ATPases, ATP7A, and ATP7B [15]. When the ion channel is damaged, it may cause a buildup of intracellular copper. The excessive copper binds to mitochondrial proteins, leading to cuproptosis [16]. Specific copper ionophores (elesclomol, disulfiram, and NSC319726) have been found to promote an increase in intracellular copper levels [1]. Among them, elesclomol is identified as a potent inducer of cuproptosis due to its strong binding affinity to copper [1]. It has been used as an anticancer agent that targets the mitochondrial metabolism, resulting in the cuproptosis of various human cancer cell lines [17]. In the genetic models of copper deficiency in nematode and mouse, it was also found that elesclomol could rescue the copper deficiency phenotypes [18].

Copper-related cellular damage is accompanied by changes in cell and mitochondrial morphology. Intracellular copper accumulation leads to the disruption of the endoplasmic reticulum and mitochondrial structure, as well as a reduction in the inner mitochondrial membrane and the production of large vesicles in *Danio rerio* [19]. Copper may also regulate the phagolysosome system to enhance the antimicrobial ability of oyster hemocytes with the assistance of mitoROS [13]. The extent of cell membrane rupture is also further exacerbated with prolonged treatment with elesclomol in human cells [3]. The observed morphological alterations are associated with the increased oxidative stress response that occurs during cuproptosis. The accumulation of intracellular copper can directly stimulate the production of ROS and lead to cell membrane rupture. Furthermore, abnormalities in mitochondria-associated proteins and proteotoxic stress during cuproptosis contribute to disruption in the intracellular energy metabolism and the subsequent accumulation of ROS, ultimately resulting in cellular rupture [6]. The main evidence for cuproptosis comes from mammals, while it is still largely unknown in non-mammalian vertebrates and invertebrates.

The Pacific oyster, *Crassostrea gigas*, as an important worldwide cultivated bivalve, has important economic and ecological values [20]. Bivalves have an enrichment effect on heavy metals, and the copper content in the soft tissue of oysters could be up to 1.9% of the dry weight of the oysters [14]. Oyster hemocytes are essential for transporting and detoxifying metals and copper exposure can increase the glutathione and heat shock proteins levels in hemocytes [21]. The potential role of hemocytes in oyster copper migration is also demonstrated by the discovery of copper ion-containing hemocytes between the gills and mantle tissues of contaminated oysters [22]. In the present study, the main cuproptosis-related genes, *Cg*FDX1 and *Cg*DLAT, were identified from the oyster *C. gigas* and the phenotypic characteristics and potential activation mechanism of cuproptosis were revealed in hemocytes after elesclomol treatment.

## 2. Materials and Methods

### 2.1. Animals

The Pacific oysters *C. gigas* (12–16 cm each in shell length), were collected from a local farm in Dalian, Liaoning, China. They were cultured in aerated seawater at 15 ± 2 °C for at least one week before the experiments. All the experiments were performed following the animal ethics guidelines approved by the Ethics Committee of Dalian Ocean University.

### 2.2. Elesclomol Treatment and Sample Collection

Ninety-six oysters were evenly divided into two groups: the DMSO-treated group and the elesclomol-treated group. Elesclomol (MedChemExpress, Monmouth Junction, NJ, USA, STA-4783) was dissolved in a solution composed of sterile seawater (contains 9.1 µg L^−1^ of copper) and L-15 Medium (1:1 in volume). The hemolymph, about 5 mL, was extracted from the sinus of each of three oysters and centrifugated at 600× *g* for 10 min at 4 °C to obtain about 1 × 10^6^ hemocytes. Hemocytes from 16 oysters were used as one sample in each group and for a total of three samples. The obtained samples were used for the detection of indicators and the verification of related gene expression. Various tissues including hepatopancreas, gonad, adductor muscle, labial palp, mantle, gills, and hemocytes, were collected from nine untreated oysters. Tissues from three oysters were pooled together as one sample, and for a total of three samples. Each sample, containing 500 μL TRIzol reagent (Thermo Fisher Scientific, Waltham, MA, USA), was stored at −80 °C for the subsequent RNA extraction that was then subjected to further analysis of *CgFDX1* and *CgDLAT* mRNA transcripts.

### 2.3. RNA Extraction, cDNA Synthesis and Sequence Analysis

Total RNA was immediately extracted using TRIzol reagent (Thermo Fisher Scientific) and the first strand of cDNA was synthesizedusing total RNA (treated with DNase I) as template and oligo dT-adaptor as primers (TransGen Biotech, Beijing, China) according to the manufacturer’s protocol [23]. The sequences of *CgFDX1* (XM_052855918.1) and *CgDLAT* (XM_052817156.1) transcripts were acquired from NCBI database (https://www.ncbi.nlm.nih.gov/ accessed on 1 April 2024) and the primers of *CgFDX1* and *CgDLAT* (Table 1) were designed to clone the full-length cDNA sequence of *CgFDX1* and *CgDLAT*, respectively. SMART online software (http://smart.embl-heidelberg.de/ accessed on 12 April 2024) was used to predict the conserved domains of *Cg*FDX1 and *Cg*DLAT. Multiple sequence alignment was generated by DNAMAN 6.0 program. The phylogenetic tree was constructed based on the amino acid sequences of FDX1s and DLATs from different species using the MEGA X (Version 10.2.6) with the neighbor-joining method, respectively [24].

### 2.4. Real-Time Quantitative PCR (RT-qPCR) Analysis of CgFDX1 and CgDLAT

The fragments of elongation factor from *C. gigas* (*CgEF*, NM_001305313) amplified with primers *CgEF*-RT-F/-R (Table 1) were employed as reference. The transcripts of *CgFDX1* and *CgDLAT* in hemocytes were detected after elesclomol treatment. The detailed steps of RT-qPCR procedures were according to the previous report [25]. The obtained data were evaluated using the 2^−ΔΔCT^ [26] and statistically analyzed with *t*-test. Significant differences were accepted at *p* < 0.05.

### 2.5. The Cell Mortality After Elesclomol Treatment

Hemocytes were enriched by centrifugation at 600× *g*, 4 °C for 10 min, and then divided into three portions. The first portion of the collected hemocytes was suspended in the solution provided by Trypan Blue Staining Cell Viability Assay Kit (Beyotime Biotechnology, Shanghai, China, C0011) and stained with the addition of an equal volume of Trypan blue solution to evaluate cell mortality under microscope. Ten microliters of cell suspension were drawn at each time point and cell mortality was calculated using a hemocytometer under the light microscope. The second portion of the collected hemocytes was re-suspended after being mixed with an anticoagulant, and the cell morphological characteristics were observed under the light microscope (ZEISS, Oberkochen, Baden-Württemberg, Germany, Axio Imager A2) [27]. The third portion was subjected to the CCK-8 assay to detect hemocyte viability. Hemocytes were seeded into 96-well plates after elesclomol treatment. Then, 10 µL of CCK-8 reagent (Jiancheng, Nanjing, China, G021-1-1) was added to each well and incubated at 18 °C for 2 h. Cell viability was assessed by measuring the absorbance at 450 nm using a microplate reader [28].

### 2.6. Contents of Copper, MDA, Pyruvic Acid, Citric Acid and Reactive Oxygen Species (ROS) After Elesclomol Treatment

The contents of copper, MDA, pyruvic acid, citric acid, and ROS in hemocytes after elesclomol treatment were determined using copper assay kit (Jiancheng, E010-1-1), MDA assay kit (Jiancheng, A003-1-2), pyruvic acid assay kit (Jiancheng, A081-1-1), citric acid assay kit (Solarbio, Beijing, China, BC2155) and ROS assay kit (Beyotime Biotechnology, Shanghai, China,, S0033S) according to the manufacturers’ instructions. The copper content was estimated by the complexometric colorimetric method; the pyruvate acid content was measured by the colorimetric method; the citrate acid was determined on the basis of the spectrophotometer method; the quantitative measurement of MDA was based on the reaction of MDA and thiobarbituric acid (TBA); the principle of ROS detection is mainly based on the properties of the fluorescent probe DCFH-DA. Hemocytes were homogenized at low temperatures, and then centrifuged at 3500× *g*, 4 °C for 10 min. The supernatant was collected for protein quantification as well as for the determination of copper, MDA, pyruvic acid, and citric acid. The concentration of total protein in the supernatant was quantified by bicinchoninic acid (BCA) method with the total protein assay kit (Jiancheng, A045-3-2).

### 2.7. Observation of Mitochondrial Morphology

Transmission electron microscopy (TEM) was used to observe the morphology of hemocyte mitochondria after elesclomol treatment. Hemocytes were collected by centrifugation at 600× *g*, 4 °C for 10 min and then fixed with 2.5% glutaraldehyde for 6 h. Treated hemocytes were dehydrated under ethanol (30%, 50%, 70%, 80%, 90%, and 95% for 15 min each), and then stored in 100% ethanol overnight at 4 °C for 24 h. The prepared samples were observed under TEM (Standard Testing Group Co., Ltd, Qingdao, China).

### 2.8. Observation of Cell Morphology

Hemocytes from elesclomol treatment were used for cell morphology observation, and those from the DMSO group were used as control. The treated hemocytes were collected by centrifugation at 600× *g*, 4 °C for 10 min and supernatant was discarded. Hemocytes were washed three times with phosphate buffered saline (PBS). Then, hemocytes were resuspended using 1:1 mixture of anticoagulant and L-15 mixed salt. A volume of 10 µL of hemocyte suspension was dropped on a poly-L-lysine-coated slide and incubated at room temperature for 4 h. The morphology of the prepared samples was observed under the light microscope. For the scanning electron microscopy analysis, samples were firstly incubated with 5% glutaraldehyde at 24 °C for 4 h. After rinsing twice with 0.1 M cacodylate, the larvae were dehydrated with graded acetone, and coated with gold. They were observed under scanning electron microscope (Hitachi High-Tech, Shanghai, China, SU8200) [29].

### 2.9. Immunocytochemical Assay of CgDLAT and CgFDX1

Immunocytochemical assay was performed to observe the subcellular localization of *Cg*DLAT and *Cg*FDX1 in hemocytes at 2 h after elesclomol treatment with DMSO as control. Hemolymph was fixed with 1 mL of a mixture containing an anticoagulant (510 mM NaCl, 100 mM glucose, 200 mM citric acid, 30 mM sodium citrate, 10 mM EDTA·2Na, pH 7.4) (1:1 in volume) and deposited on glass slides for 2 h. Then, 200 μL 4% paraformaldehyde (PFA) was added and fixed for 30 min. Then, samples on the glass slides were blocked with 3% bovine serum albumin (BSA) at room temperature for 2 h and incubated with anti*-Cg*DLAT or anti*-Cg*FDX1 antibody (1:1000 in 3% BSA) at 4 °C overnight. After washing three times with PBS solution containing 0.1% Tween 20 (PBST), samples were incubated with Alexa Fluor 488-labeled goat anti-mouse antibody (Beyotime, diluted 1:1000 (*v*/*v*) in 3% BSA) at 37 °C in dark for 1 h. Then, hemocytes were incubated with 1 mL of 100 nM Mito-Tracker Red CMXRos solution (Beyotime, C1049) for 30 min. Eventually, hemocytes were incubated with antifade mounting medium containing 4′,6-diamidino-2-phenylindole (DAPI) (Beyotime, P0131). Treated slides were stored in glycerin and observed under a laser confocal microscope (LSM 800, ZEISS).

### 2.10. Flow Cytometry (FCM) Analysis of Hemocytes

Collected untreated hemocytes were fixed in 4% PFA for 15 min to preserve the integrity of cell morphology. In order to assess mitochondrial damage, the expressions of mitoSOX (Beyotime, S0061S) and mitochondrial membrane potential (JC-1) (Beyotime, C2006) were examined separately by FCM. Following sample collection, a portion of cells was resuspended by adding 1 mL of mitoSOX red staining solution. Then, cells were incubated in a light-proof environment at 37 °C for 30 min. Subsequently, they were centrifuged at 600× *g* 4 °C for 5 min to pellet the cells, and the supernatant was discarded. Then, cells were washed twice with PBST. One milliliter PBST was added to resuspend cells and the other part of the cells was resuspended by adding 1 mL JC-1 staining solution. After incubation at 37 °C for 20 min, cells were centrifuged at 600× *g* for 5 min at 4 °C. The cells were washed twice with JC-1 staining buffer (1×) and then resuspended by adding 1 mL of JC-1 staining buffer (1×). The signals of treated hemocytes were analyzed by FCM (BD FACSAria™ II flow cytometer, Franklin Lakes, NJ, USA). Moreover, a total of 10,000 intact hemocytes were collected as one sample and there were three replicates. The values of the fluorescence intensity in each sample were recorded using the flow cytometer [30].

### 2.11. Preparation of Antibody and Western Blot Assay

The peptide antigen of *Cg*DLAT (XM_011414149.3) (DG peptides Co., Ltd., Wuhan, China) was synthesized by 100 μL polypeptide (1 mg mL^−1^) dissolved in Tris Buffered Saline (TBS) and 100 μL adjuvant was mixed to form a stabilizing emulsion to treat 6-week-old mice. As the amino acid sequence of *Cg*FDX1 had higher conservation with that of *Hs*FDX1, the specificity of the commercial FDX1 antibody (Solarbio, K006063P) was verified in hemocyte lysates using Western blot assay. Total hemocyte lysates were separated by 15% SDS-PAGE and then transferred onto nitrocellulose membranes. The membrane was blocked by 5% skimmed milk (100 mL TBS with 0.1% Tween-20 (TBST), 5 g skimmed milk) at room temperature for 2 h, and then the membrane was incubated with anti-*Cg*DLAT and anti-*Cg*FDX1 antibody as primary antibody at 4 °C overnight, respectively. After washing with TBST three times, alkaline phosphatase-conjugated goat anti-rabbit IgG (Beyotime, A0208) as secondary antibody was diluted 1:1000 in TBST and incubated with the membranes at room temperature for 2 h. After washing with TBST three times, membranes were immersed in the reaction mixture (1:1 mixture of BeyoECL Moon A and B) in the dark for 1 min. The band signals were imaged by Amersham Imager 600 (GE Healthcare, Chicago, IL, USA) [31]. Another portion of hemocytes was extracted by immunoprecipitation methods for BeyoGel™ Plus Precast PAGE Gel (4 to 10%) for Tris-Gly System (Beyotime, P0465S) at 80 V for 3 h. The gel was observed and pictured by Amersham Imager 600 (GE Healthcare) [32].

### 2.12. Statistical Analysis

All statistical analyses were carried out with data reported as means ± SD. One-way ANOVA and post hoc multiple comparisons were conducted using SPSS 22.0. Differences were considered significant at *p* < 0.05 (*), highly significant at ** *p* < 0.01, and extremely significant at *** p < 0.001.

## 3. Results

### 3.1. Sequence Characteristics and Phylogenetic Relationship of CgFDX1 and CgDLAT

**Figure 1:**
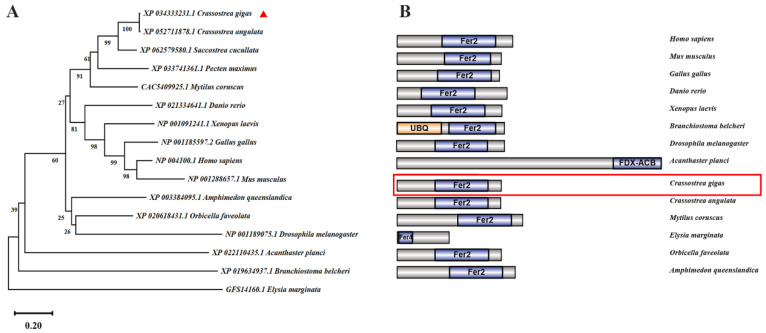
Bioinformatics analysis of *Cg*FDX1. (**A**) Phylogenetic analysis of FDX1s. The neighbor-joining phylogenetic tree based on the sequences of FDX1s from different species by MEGA X software. The number at the forks indicates the bootstrap. (**B**) The structure domain of FDX1s predicted by SMART and plotting analysis by IBS 1.0.3 software. Fer2: ferritin 2; UBQ: ubiquitin structural domain; FDX-ACB: ferredoxin-fold anticodon binding domain; Fer4: ferritin 4. *Cg*FDX1 was marked with red triangle (**A**) and red box (**B**).

**Figure 2:**
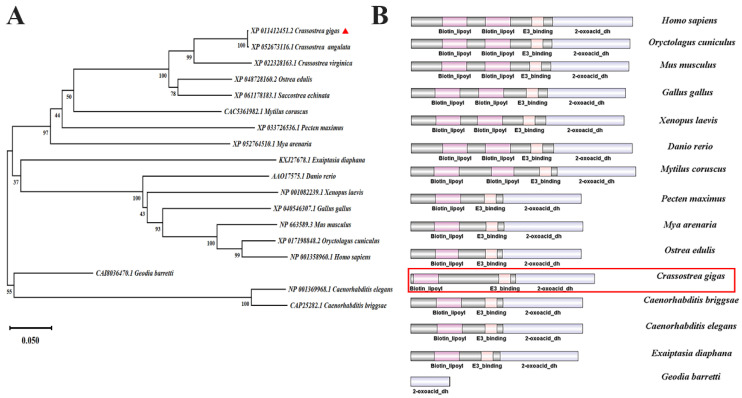
Bioinformatics analysis of *Cg*DLAT. (**A**) Phylogenetic analysis of DLATs. The neighbor-joining phylogenetic tree based on the sequences of DLATs from different species by MEGA X software. The number at the forks indicates the bootstrap. (**B**) The structure domain of DLATs predicted by SMART and plotting analysis by IBS 1.0.3 software. Biotin_lipoyl: biotin and lipoic acid attachment; E3-binding: E3 ubiquitin ligase binding; 2-oxoacid_dh: 2-oxoacid dehydrogenases acyltransferase domain. *Cg*DLAT was marked with red triangle (**A**) and red box (**B**).

*FDX1*s and *DLAT*s were present in both vertebrates and invertebrates, according to the mining of the genomes. The amino acid sequences of FDX1s and DLATs were employed to construct the phylogenetic analysis in order to elucidate their phylogenetic relationship. *Cg*FDX1 was closely clustered with *Ca*FDX1 from *Crassostrea angulata* and then gathered with FDX1s from *Mytilus coruscus* and *Orbicella faveolata*. Those from vertebrates, such as *Homo sapiens*, *Mus musculus*, *Gallus gallus*, *Xenopus laevis*, and *Danio rerio* were clustered together (Figure 1A). According to the phylogenetic tree of DLATs, *Cg*DLAT was closely clustered with *Oe*DLAT from *Ostrea edulis* and then gathered with *M. coruscus*, *Meloidogyne arenaria*, and *Pecten maximus*. Those from vertebrates, such as *H. sapiens*, *M. musculus*, *G. gallus*, *X. laevis* and *D. rerio* were clustered together (Figure 2A). The complete cDNA sequence of *CgFDX1* was of 1480 bp and the open reading frame (ORF) was of 501 bp encoding a polypeptide of 166 amino acid residues with a predicted molecular mass of 18.30 kDa and a theoretical isoelectric point of 5.38. In species of vertebrates and invertebrates (except for *Drosophila*), FDX1 generally contained a classical ferritin 2 (Fer2) domain (Figure 1B). The full-length cDNA sequence of *CgDLAT* was of 2005 bp, and the ORF was of 1629 bp encoding a polypeptide of 542 amino acid residues with a predicted molecular mass of 58.46 kDa and a theoretical isoelectric point of 7.48. In different species of vertebrates and invertebrates (except for sponges), DLAT normally contained one or two biotin and lipoic acid attachment domains (Biotin_lipoyl), an E3 ubiquitin ligase binding domain (E3-binding), and a 2-oxoacid dehydrogenases acyltransferase domain (2-oxoacid_dh). DLAT in sponges only contained a 2-oxoacid_dh domain (Figure 2B).

### 3.2. Expression Pattern of CgFDX1 and CgDLAT mRNA in Different Tissues

**Figure 3:**
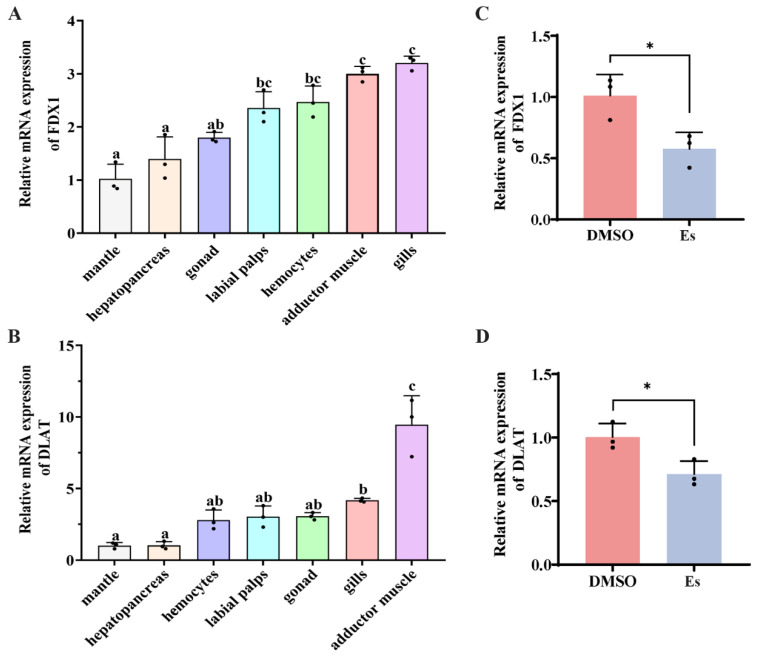
The mRNA transcripts of *CgFDX1* and *CgDLAT*. (**A**,**B**) The mRNA transcripts of *CgFDX1* and *CgDLAT* in different tissues. (**C**,**D**) The mRNA transcripts of *CgFDX1* and *CgDLAT* after elesclomol treatment. DMSO treatment was employed as the control. The vertical bars are shown as means ± S.D. (*n* = 3). The different letters show that there exist significant differences compared with other groups (*p* < 0.05, one-way ANOVA). Asterisks indicate significant differences (* *p* < 0.05).

The mRNA transcripts of *CgFDX1* were detected in hemocytes, gonad, mantle, labial palp, gills, adductor muscle, and hepatopancreas with relatively higher levels in the gills, adductor muscle, and hemocytes (3.2-fold, 3-fold, and 2.47-fold more than in the mantle, respectively) (Figure 3A). The mRNA transcripts of *CgDLAT* were also examined in all the tested tissues with a relatively higher amount in the gills, adductor muscle, and gonad (9.46-fold, 4.17-fold and 3.05-fold more than in the mantle, respectively) (Figure 3B).

### 3.3. Expressions of CgFDX1 and CgDLAT After Elesclomol Treatment

**Figure 4:**
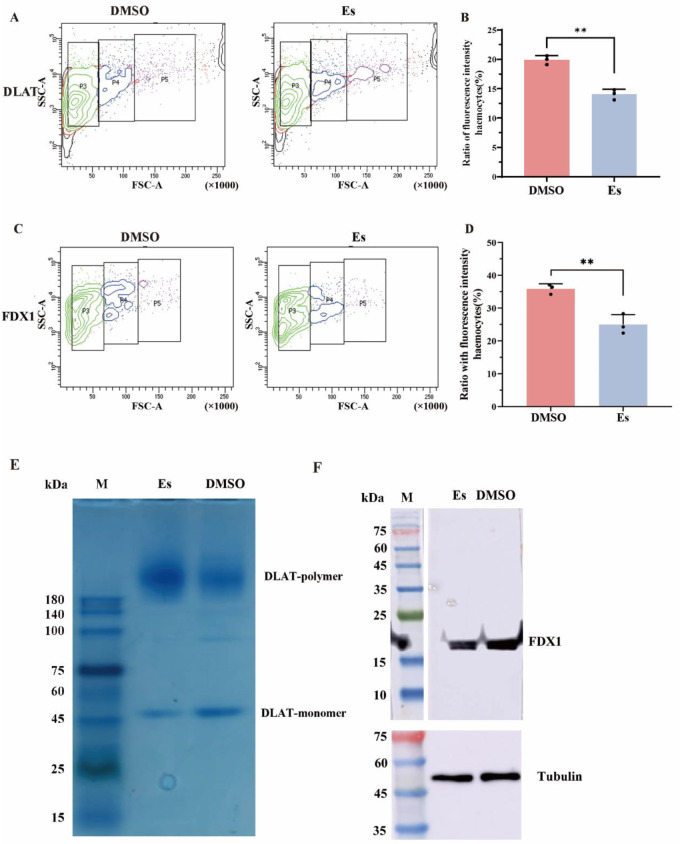
Protein distribution and expressions of *Cg*FDX1 and *Cg*DLAT after elesclomol treatment. (**A**–**D**) The protein signal intensities of *Cg*FDX1 and *Cg*DLAT in hemocytes after elesclomol treatment. Anti-*Cg*DLAT and anti-*Cg*FDX1 conjugated to Alexa-fluor 488 is shown in green fluorescence signal. P3, P4, and P5: granular cells (green), semi-granular cells (blue) and agranulocyte cells (purple). (**E**) After immunoprecipitation with anti-*Cg*DLAT from hemocyte lysates, bound proteins were separated by NATIVE-PAGE followed Coomassie Staining after elesclomol treatment. (**F**) Western blot analysis of *Cg*FDX1 after elesclomol treatment. Lane M: standard protein molecular weight marker, Lane Es: elesclomol treatment group, Lane DMSO: DMSO treatment group. β-Tubulin was employed as a loading control. The vertical bars are shown as mean ± S.D. (*n* = 3). Asterisks indicate significant differences (** *p* < 0.01).

The mRNA transcripts of *CgFDX1* and *CgDLAT* in hemocytes after elesclomol treatment were examined by RT-qPCR with *CgEF* as internal control. The mRNA transcripts of *CgFDX1* and *CgDLAT* in hemocytes decreased significantly after elesclomol treatment, which was 0.56-fold and 0.7-fold (*p* < 0.05) more than in the DMSO treatment (Figure 3C,D). The distribution of *Cg*FDX1 and *Cg*DLAT in hemocytes after elesclomol treatment was analyzed using FCM assay. The fluorescence intensities of *Cg*FDX1 and *Cg*DLAT in elesclomol treatment were 0.69-fold (*p* < 0.01) and 0.71-fold (*p* < 0.01) greater than in the DMSO treatment group (Figure 4A–D). The specificity of *Cg*DLAT and *Cg*FDX1 antibody was confirmed by Western blotting assay. A single band about 18.4 kDa with the high specificity was revealed, which was identical to the prediction of molecular mass of CgFDX1. And the single band about 58.46 kDa with the high specificity also was found to match the predicted molecular mass of CgDLAT (Figure S1A,B). The expression of *Cg*FDX1 protein decreased significantly after elesclomol treatment and the expression of *Cg*DLAT monomer decreased while the expression of *Cg*DLAT polymer increased significantly (Figure 4E,F and Figure S2A–C).

### 3.4. Colocalization of CgFDX1 and CgDLAT with Mitochondria After Elesclomol Treatment

**Figure 5:**
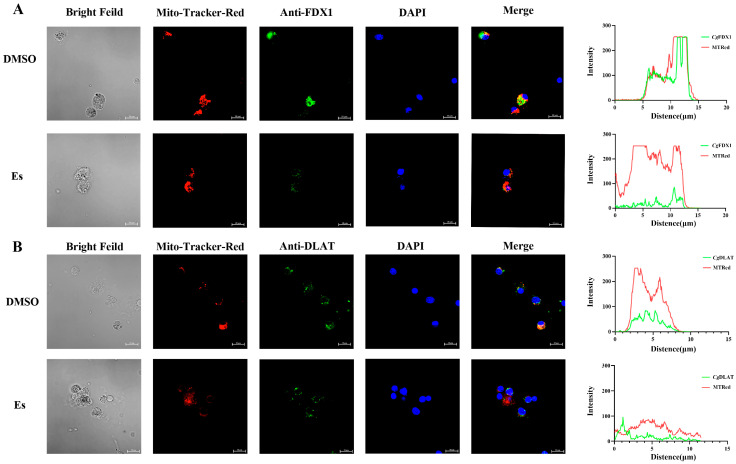
The colocalization of *Cg*FDX1 and *Cg*DLAT with mitochondria in hemocytes after elesclomol treatment. (**A**) The colocalization of *Cg*FDX1 with mitochondria in hemocytes after elesclomol treatment. (**B**) The colocalization of *Cg*DLAT with mitochondria in hemocytes after elesclomol treatment.

To visualize the colocalization of *Cg*FDX1 and *Cg*DLAT with mitochondria, *Cg*FDX1 or *Cg*DLAT conjugated with Alexa Flour 488 is shown in green, and the mitochondria labelled with Mito-Tracker Red is in red. Nuclei were stained with DAPI in blue. The signals of *Cg*FDX1 (green) and the mitochondria (red) were mainly in the hemocyte cytosol in the DMSO treatment and the elesclomol treatment. Colocalization signals of *Cg*FDX1 (green) and mitochondria (red) were reduced in the hemocytes after elesclomol treatment (Figure 5A). Co-localization signals of *Cg*DLAT (green) and mitochondria (red) in hemocytes were also attenuated after elesclomol treatment, and the distinct punctate aggregation fluorescence of *Cg*DLAT was observed in the hemocytes (Figure 5B).

### 3.5. The Contents of Copper, MDA, Citric Acid and Pyruvic Acid After Elesclomol Treatment

**Figure 6:**
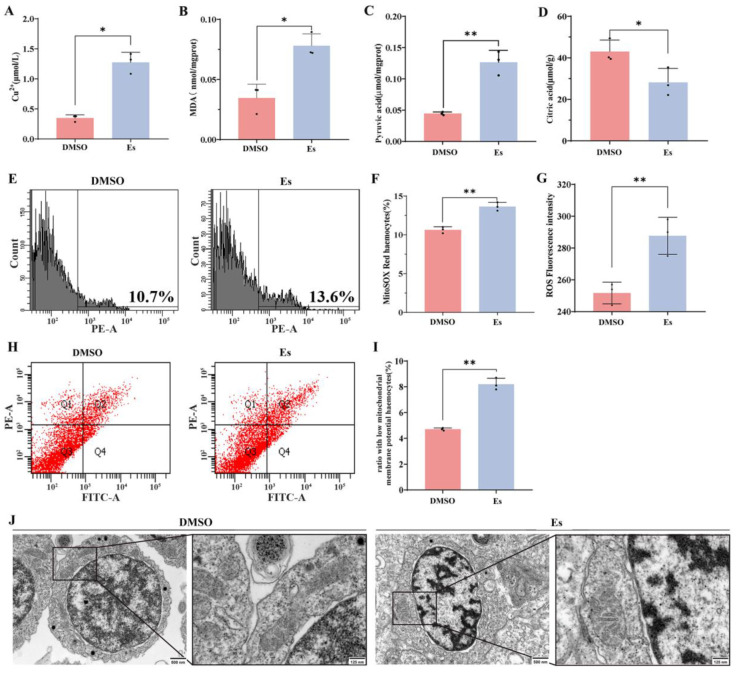
Copper contents and mitochondrial status in hemocytes after elesclomol treatment. (**A**–**D**) Contents of copper, MDA, pyruvic acid, and citric acid in hemocytes after elesclomol treatment. (**E**,**F**) The mitoSOX red fluorescence intensity in hemocytes after elesclomol treatment. (**G**) Fluorescence intensity of ROS probe after elesclomol treatment. (**H**,**I**) Ratio with low mitochondrial membrane potential in hemocytes after elesclomol treatment. (**J**) Mitochondrial morphology of hemocytes after elesclomol treatment. Mitochondrial morphology of hemocytes in elesclomol and DMSO treatment under TEM. Vertical bars represent the mean ± S.D. (*n* = 3). Asterisks indicate significant differences (* *p* < 0.05, ** *p* < 0.01).

The copper content in hemocytes after elesclomol treatment increased significantly: it was 3.68-fold (*p* < 0.05) higher than in the DMSO treatment group (Figure 6A and Figure S1C). The MDA content in the hemocytes after elesclomol treatment also increased significantly: it was 2.26-fold (*p* < 0.05) higher than in the DMSO treatment group (Figure 6B). The contents of pyruvic acid and citric acid in the hemocytes of the elesclomol treatment group increased significantly: they were 2.84-fold (*p* < 0.01) and citric acid decreased significantly: it was 0.65-fold (*p* < 0.05) that in DMSO treatment. (Figure 6C,D).

### 3.6. FCM Analysis of Hemocytes After Elesclomol Treatment

The effects of mitochondria in hemocytes of elesclomol treatment were evaluated with mitochondrial superoxide (mitoSOX) assay kit, ROS detection kit, and mitochondrial membrane potential assay kit. The percentage of mitoSOX Red in the hemocytes of the elesclomol treatment group increased significantly: it was 1.28-fold (*p* < 0.01) greater than in the DMSO treatment group (Figure 6E,F). The average fluorescence intensity of ROS in the hemocytes of the elesclomol treatment group increased significantly: it was 1.14-fold (*p* < 0.01) higher than in the DMSO treatment group (Figure 6G). The percentage of low potential in hemocytes with the elesclomol treatment increased significantly: it was 1.74-fold (*p* < 0.01) greater than in the DMSO treatment group (Figure 6H,I).

### 3.7. Morphology of Hemocytes and the Morphology of Mitochondria in Hemocytes After Elesclomol Treatment

**Figure 7:**
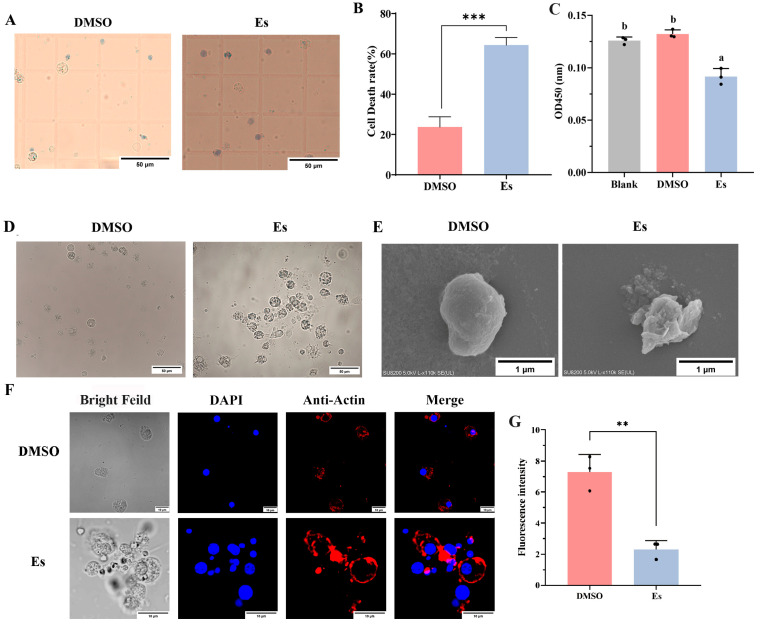
The morphology and mortality of hemocytes after elesclomol treatment. (**A**,**B**) Cell mortality of hemocytes after elesclomol treatment. (**C**) The viability of hemocytes after elesclomol treatment was tested by CCK-8 assay. (**D**,**E**) Morphology hemocytes by 40× microscopy and SEM after elesclomol treatment. (**F**) Skeletal structure of hemocytes after elesclomol treatment observed by confocal microscopy. (**G**) Statistical analysis of fluorescence intensity of cytoskeleton in hemocytesusing ImageJ software (version 1.51k). Vertical bars represent the mean ± S.D. (*n* = 3). The different letters show that there exist significant differences compared with other groups (*p* < 0.05, one-way ANOVA). Asterisks indicate significant differences (** *p* < 0.01; *** *p* < 0.001).

The morphology of the hemocytes was observed by microscope. The ultrastructure of mitochondria in hemocytes after elesclomol treatment was altered, with the mitochondrial cristae becoming sparse and detached (Figure 6J). Trypan blue staining showed that the death rate of hemocytes treated with elesclomol was significantly higher (*p* < 0.05) than that of the DMSO treatment group (Figure 7A,B). The viability of hemocytes was significantly reduced after elesclomol treatment: it was 0.69-fold lower than in the DMSO treatment. There was no significant change in the DMSO treatment and blank groups (Figure 7C). The hemocytes in the DMSO treatment group were observed with complete and smooth cell membrane and the cells were relatively independent. As compared with DMSO treatment, some hemocytes after elesclomol treatment showed obvious cell membrane shrinkage, intercellular adhesion (Figure 7D,E). Actin-Tracker Red-555 staining showed that the internal structure of the hemocytes significantly changed after elesclomol treatment and the staining fluorescence of red filamentous morphology was weakened significantly: it was 0.32-fold (*p* < 0.01) weaker than with DMSO treatment (Figure 7F,G).

## 4. Discussion

Cuproptosis is a recently identified form of copper-dependent programmed cell death [33,34]. The cytotoxicity of elesclomol is significantly dependent on its ability to transport extracellular copper, which plays a crucial role in the process of cuproptosis [17]. Elesclomol transports copper to mitochondria, restores oxidase enzyme function and alleviates deficiency symptoms in models like *Saccharomyces cerevisiae*, *D. rerio*, and *M. musculus* [35]. If it has a similar function to induce cell cuproptosis in other species, this is still unknown. In the present study, characteristic alterations associated with cuproptosis were detected in the hemocytes of *C. gigas* after elesclomol treatment, which were analogous to the cuproptosis phenomena observed in higher animals.

Genome-wide CRISPR/Cas9 screening identified the genes related to cuproptosis induced by copper ionophores, among which, the key genes involved in the main mechanism include *FDX1* and *DLAT* [1]. FDX1 serves as a crucial molecule directly targeted by elesclomol that encodes an enzyme responsible for the reduction of Cu^2+^ to Cu^+^ [36,37]. It has been evidenced that the depletion of FDX1 gives rise to a decline in the level of lipoyl synthase, thereby resulting in cuproptosis [38]. DLAT is situated in the inner mitochondrial membrane and represents the E2 subunit of the pyruvate dehydrogenase complex. It exerts a function in mitochondrial glucose metabolism, converting the acetyl group generated by the oxidative decarboxylation of pyruvate into coenzyme A [39]. In the present study, phylogenetic analysis suggested that *Cg*FDX1 was firstly clustered with *Ca*FDX1 from *C. angulata* and *Cg*DLAT was firstly clustered with *Oe*DLAT from *Ostrea edulis*, and they were categorized within the spiralia branch, suggesting their relatively conserved evolutionary relationships. *FDX1* and *DLAT* were systematically screened and analyzed in the genomes of species from different metazoan phyla to provide a reference for validating the possibility that cuproptosis occurs in oysters. In humans, FDX1 contained an Fer2 domain and DLAT contained two biotin_lipoyl domains, an E3-binding domain and a 2-oxoacid_dh domain. *FDX1* and *DLAT* were also present in fish. In invertebrates, both genes were present in representative species. *FDX1* and *DLAT* firstly appeared in two sponges (*Amphimedon queenslandica* and *Geodia barretti*) and then in bivalves and even higher species, *FDX1* and *DLAT* were preliminarily determined to be evolutionarily conserved by analysis. On these findings, it was hypothesized that the mechanism of cuproptosis might be present in invertebrates. Additionally, *FDX1* and *DLAT* are expressed in various tissues of vertebrates and invertebrates. In mice, the highest level of mRNA transcripts of *FDX1* were found in adrenal and ovary tissue, and the highest mRNA transcripts of *DLAT* were found in heart and mammary gland [40,41]. In invertebrates, the systematic study of the organization and distribution of *FDX1* and *DLAT* has not yet been investigated. The detection of tissue distribution at the mRNA transcripts might offer a more comprehensive insight into the physiological responses of oysters to copper stress, encompassing immune reactions, and metabolic alterations [42]. In the present study, the level of *CgFDX1* and *CgDLAT* transcripts could be detected in all the tested tissues, and their transcripts were higher in adductor muscle, hemolymph, and gills. The characterization of *Cg*FDX1 and *Cg*DLAT in oysters indicated that FDX1 and DLAT exhibited significant conservation in terms of structure, evolution, and distribution. The transcripts of *CgFDX1* and *CgDLAT* in various tissues suggested that cuproptosis might play a critical role in these tissues. They were relatively higher in gills and mantle, suggested the important function of cuproptosis in these tissues.

Elesclomol has been extensively employed in cancer therapy owing to its effects on mitochondrial metabolism. Recent research has revealed that elesclomol potentially possesses therapeutic applications in cancer cells through the induction of cuproptosis [17]. A specific concentration of elesclomol facilitates the transport of copper into cells, resulting in copper accumulation and subsequent induction of cuproptosis [1]. However, if it has a similar function to induce cell cuproptosis in other species, this is still unknown. Cuproptosis is mainly caused by the accumulation of copper in mitochondria, resulting in impaired mitochondrial function and cell death [43]. In the present study, the release of cellular contents, the occurrence of cell rupture and adhesion, and the increase in cell death demonstrate the exacerbation of cellular damage after elesclomol treatment. Copper directly combines with lipoylated proteins in the mitochondrial TCA cycle to lead to mitochondrial damage during cuproptosis [5]. Mitochondria is the hub of the intracellular energy metabolism, exerting a crucial role in the process of cellular respiration and serving as the primary production site of ROS [44]. Oxidative stress is usually defined as an increase in intracellular ROS levels when the cell is subjected to external stress, resulting in damage to lipids, proteins, and DNA [45]. The mitochondrial membrane potential serves as a crucial indicator of the health and functional status of cells. JC-1 dye could serve as an indicator for mitochondrial membrane potential in FCM analysis [46]. In the present study, mitochondria were observed with shrunken mitochondrial membranes and cristae, the latter sometimes disappearing after elesclomol treatment. The proportion of low potential in the inner mitochondrial membrane increased significantly and the membrane potential decreased significantly, while the content of mitoSOX and ROS also increased significantly after elesclomol treatment. The above results indicate that elesclomol treatment could cause mitochondrial damage in oyster hemocytes, and the damage might be related to the occurrence of cuproptosis.

Copper accumulation and the subsequent series of cascaded reactions are the key features of cuproptosis. Excessive copper will combine with key proteins in the TCA cycle, inducing oxidative stress in cells [16]. Hemocytes could be classified into different phenotypes of biomineralized and immunoreactive cells based on cell morphology, gene expression patterns, motility and adhesion characteristics [47]. Copper may be sequestered in the lysosomes of oyster hemocytes via the transporter to mitigate their toxicity [21]. Copper exposure also induced the expression of certain membrane proteins and lipid transport activity in hemocytes, activated the lysosomal signaling pathway, facilitated the transport of intercellular lysosomes, and maintained the homeostasis and functionality of hemocytes [21]. In the present study, the copper content in hemocytes increased significantly after elesclomol treatment, suggesting that elesclomol might cause copper metabolism disorder in oysters. MDA is one of the major end products of peroxidation of polyunsaturated fatty acids within cells, and an augmentation of free radicals will result in excessive generation of MDA. The level of MDA is typically regarded as a significant biomarker of oxidative stress and antioxidant status [48]. In the present study, the content of MDA in hemocytes increased significantly after elesclomol treatment, suggested that intracellular antioxidant capacity was reduced and oxidative damage was enhanced in hemocytes after elesclomol treatment. DLAT encodes the E2 subunit of mitochondrial PDHc (pyruvate dehydrogenase complex) and is essential for its activity. The aberrant DLAT expression likely impairs PDHc function [49]. Pyruvic acid as the final product of glycolysis is an important intermediate of the amino acid and protein metabolism [50], which catabolism is affected by DLAT. In addition, citric acid is an important organic compound in the TCA cycle and plays an important role in maintaining normal and stable function. In the present study, the content of pyruvic acid increased significantly and the content of citric acid decreased significantly after elesclomol treatment, suggested that elesclomol treatment inhibits the catabolism of pyruvate acid and disrupts the normal progression of the TCA cycle. These results suggest that elesclomol treatment potentially plays a role in modulating mitochondrial toxic stress and the TCA cycle in hemocytes of *C. gigas*, which might be associated with cuproptosis.

The enrichment of copper within the cell caused by elesclomol and FDX1 eventually leads to the oligomerization of DLAT. Additionally, elesclomol binding to FDX1 inhibits Fe-S cluster protein synthesis during cuproptosis [1,51]. In the present study, the mRNA transcription of *CgFDX1* and *CgDLAT* decreased significantly in elesclomol-treated hemocytes. Furthermore, the accumulation of excessive copper leads to modifications in mitochondrial protein structures, thereby facilitating cuproptosis primarily within mitochondria. In the present study, the colocalization of *Cg*FDX1 and *Cg*DLAT with mitochondria was weakened significantly, while the fluorescence of *Cg*DLAT also appeared as punctate aggregates after elesclomol treatment. These above results suggest that *Cg*FDX1 and *Cg*DLAT participate in the death of hemocytes induced by elesclomol and the regulatory mechanisms are conserved and highly similar to cuproptosis in mammals.

## 5. Conclusions

**Figure 8:**
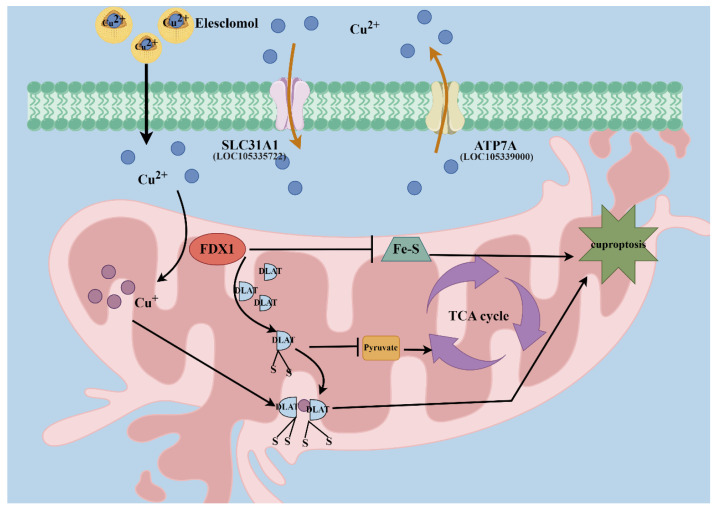
Mechanism of cuproptosis induced by elesclomol in hemocytes. Copper ionophores such as elesclomol transport copper into the intracellular compartments. Excess copper combines with mitochondrial-related proteins (FDX1 and DLAT) and leads to their functional impairment. These alterations result in mitochondrial protein toxic stress and mitochondrial dysfunction, eventually triggering hemocyte cuproptosis in oysters. Mechanism diagram created by figdraw.com (authorized for use, ID: UTUAWb24b2).

In conclusion, elesclomol could be a potential inducer to induce cuproptosis in the hemocytes of *C. gigas*. The rate of cell death in hemocytes increased significantly, while the cytoskeletal structure and mitochondrial morphology exhibited changes after elesclomol treatment. The contents of copper, MDA, pyruvic acid, and ROS increased significantly, while the content of citric acid decreased significantly after elesclomol treatment. Additionally, the mitochondrial-related indices appeared significantly changed, while both the mRNA and protein expressions of *Cg*FDX1 and *Cg*DLAT in hemocytes changed significantly after elesclomol treatment (Figure 8). These results indicate that elesclomol has the potential to trigger cuproptosis in the hemocytes of oysters, and the mechanism is highly concordant with that of cuproptosis discovered in mammals [1].

## Figures and Tables

**Table 1:** Sequences of the primers used in this study.

Primer	Sequence (5′-3′)
RT-PCR primers	
*CgFDX1*-RT-F	ATCAAAGACAAGCCCACAGACGA
*CgFDX1*-RT-R	CTTGGTAACAATCACTTGACAGCCTAAT
*CgDLAT*-RT-F	TTGTAGTCACACCTGGGGCAGAGTA
*CgDLAT*-RT-R	ACCGCTTGGCTATTGTCTTTCTCAT
*CgEF*-RT-F	AGTCACCAAGGCTGCACAGAAAG
*CgEF*-RT-R	TCCGACGTATTTCTTTGCGATGT

## Data Availability

Original data are available upon request.

## Supplementary Materials

The following supporting information can be downloaded at: https://www.mdpi.com/article/10.3390/cells14030199/s1, Figure S1: The antibody verification of CgDLAT (A) and CgFDX1 (B) in hemocytes as well as the concentration of Cu2+ in hemocytes after elesclomol treatment (C); Figure S2: The full length uncropped original western blots of CgFDX1 (A), β-Tubulin (B), CgDLAT (C).
