# Supplementary material for: The Potential Mechanism of Cuproptosis in Hemocytes of the Pacific Oyster Crassostrea gigas upon Elesclomol Treatment
Source: Cells. 2025 Jan 29;14(3):199. doi: 10.3390/cells14030199 (PMC11817986; doi:10.3390/cells14030199)
Supplement: Supplementary file 1 [file cells-14-00199-s001.zip › Supplementary materials.pdf]

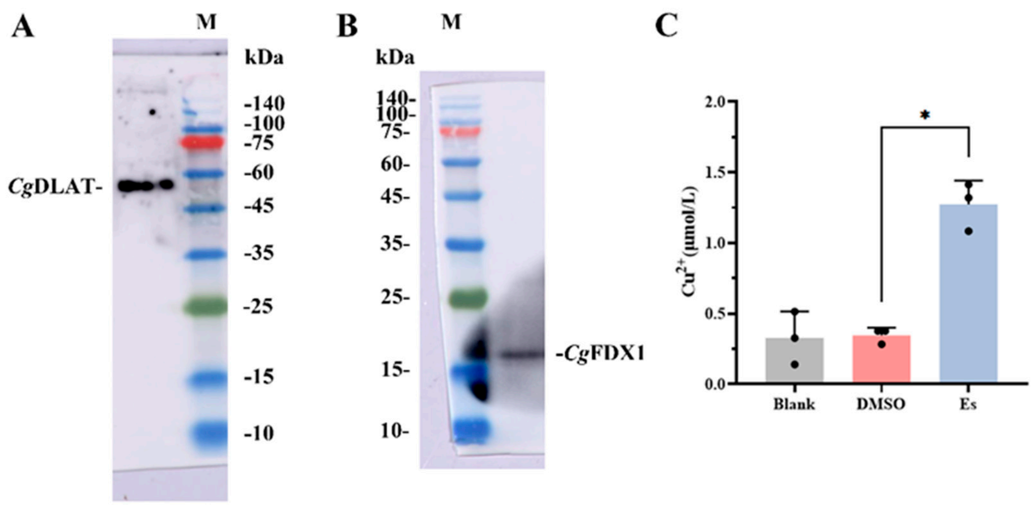

Figure S1 The antibody verification of *Cg*DLAT **(A)** and *Cg*FDX1 **(B)** in hemocytes as well as the concentration of Cu^{2+} in hemocytes after elesclomol treatment **(C)**.

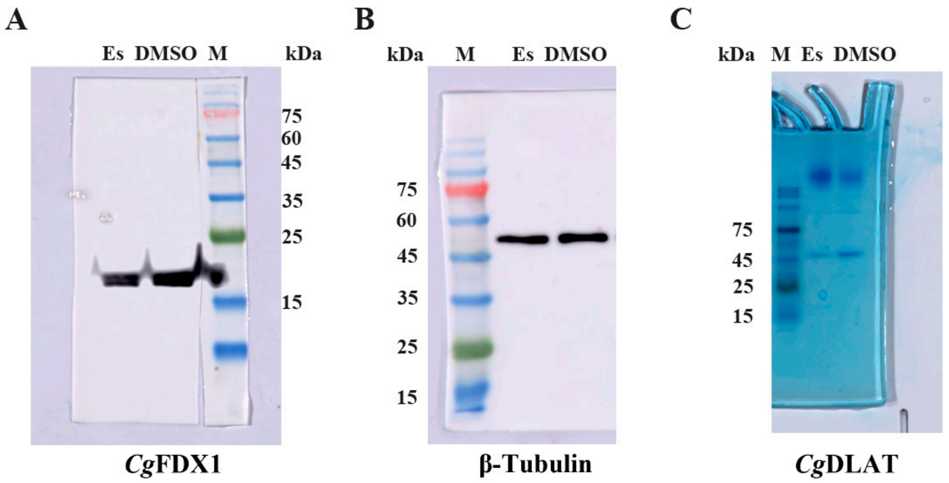

Figure S2 The full length uncropped original western blots of *Cg*FDX1 **(A)**, β-Tubulin **(B)**, *Cg*DLAT **(C)**.
