# Supplementary figures and images for: The Potential Mechanism of Cuproptosis in Hemocytes of the Pacific Oyster Crassostrea gigas upon Elesclomol Treatment
Source: Cells. 2025 Jan 29;14(3):199. doi: 10.3390/cells14030199 (PMC11817986; doi:10.3390/cells14030199)

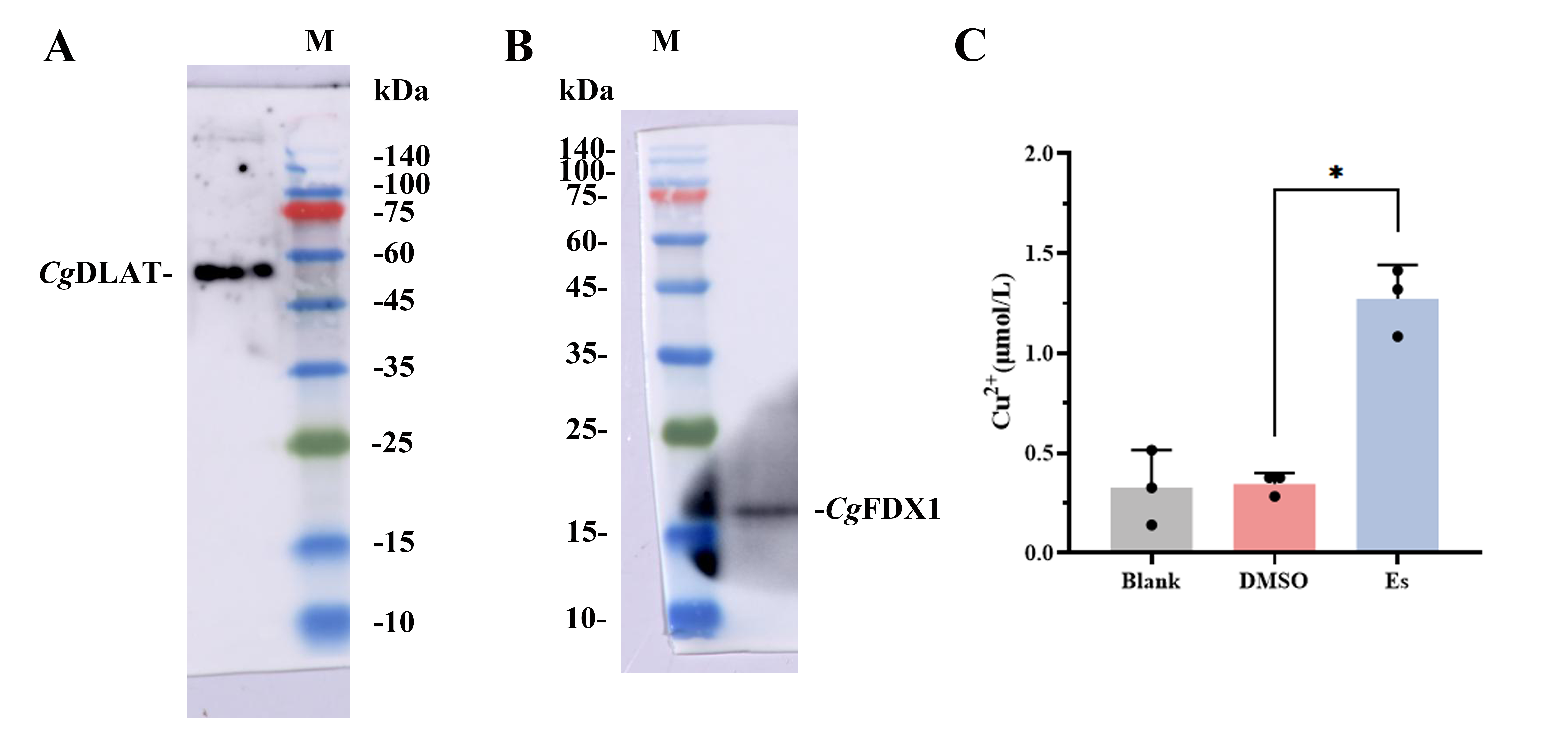

Supplement: Supplementary file 1 [file cells-14-00199-s001.zip › Figure/Figure-S1.tif]

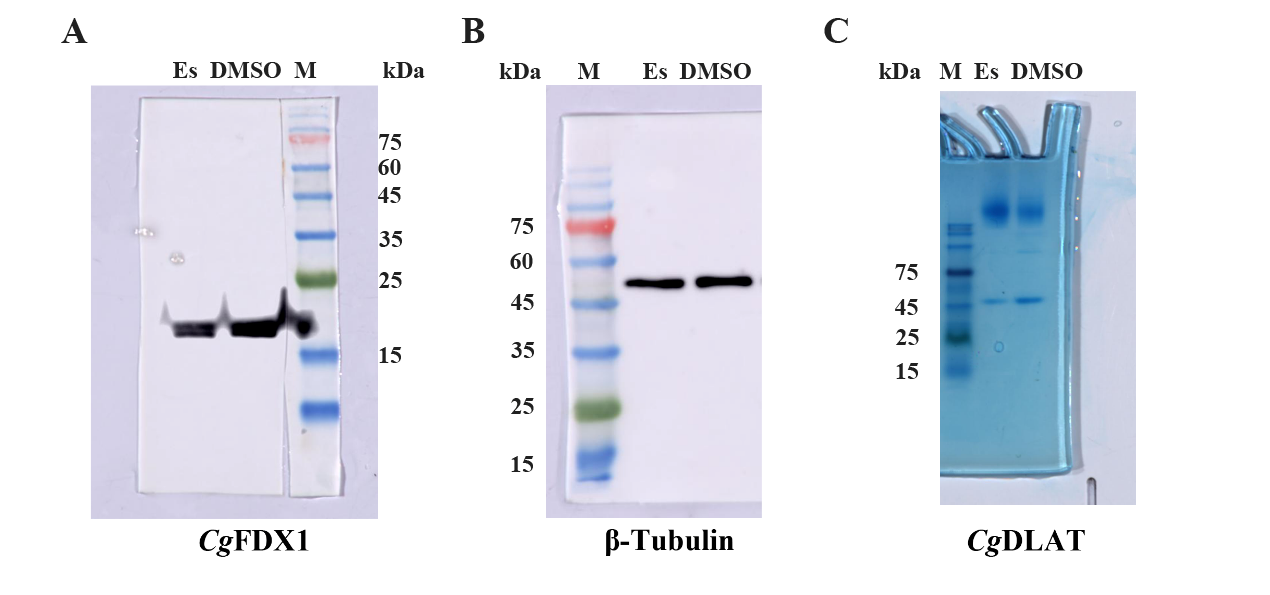

Supplement: Supplementary file 1 [file cells-14-00199-s001.zip › Figure/Figure-S2.tif]
